# The Impact of Poor Nutrient Intakes and Food Insecurity on the Psychological Distress among Community-Dwelling Middle-Aged and Older Adults during the COVID-19 Pandemic

**DOI:** 10.3390/nu13020353

**Published:** 2021-01-25

**Authors:** Nurul Fatin Malek Rivan, Hanis Mastura Yahya, Suzana Shahar, Devinder Kaur Ajit Singh, Norhayati Ibrahim, Arimi Fitri Mat Ludin, Noor Ibrahim Mohamed Sakian, Hazlina Mahadzir, Ponnusamy Subramaniam, Mohd Zul Amin Kamaruddin

**Affiliations:** 1 Nutritional Sciences Programme and Centre for Healthy Ageing and Wellness (H-CARE), Faculty of Health Sciences, Universiti Kebangsaan Malaysia, Jalan Raja Muda Abdul Aziz, Kuala Lumpur 50300, Malaysia; fatinmalek93@gmail.com; 2 Dietetics Programme and Centre for Healthy Ageing and Wellness (H-CARE), Faculty of Health Sciences, Universiti Kebangsaan Malaysia, Jalan Raja Muda Abdul Aziz, Kuala Lumpur 50300, Malaysia; suzana.shahar@ukm.edu.my; 3 Physiotherapy Programme and Centre for Healthy Ageing and Wellness (H-CARE), Universiti Kebangsaan Malaysia, Jalan Raja Muda Abdul Aziz, Kuala Lumpur 50300, Malaysia; devinder@ukm.edu.my; 4 Health Psychology Programme and Centre for Healthy Ageing and Wellness (H-CARE), Faculty of Health Sciences, Universiti Kebangsaan Malaysia, Jalan Raja Muda Abdul Aziz, Kuala Lumpur 50300, Malaysia; yatieibra@ukm.edu.my (N.I.); ponnusaami@ukm.edu.my (P.S.); 5 Biomedical Science Programme and Centre for Healthy Ageing and Wellness (H-CARE), Universiti Kebangsaan Malaysia, Jalan Raja Muda Abdul Aziz, Kuala Lumpur 50300, Malaysia; arimifitri@ukm.edu.my; 6 Occupational Therapy Programme, Centre for Healthy Ageing and Wellness (H-CARE), Faculty of Health Sciences, Universiti Kebangsaan Malaysia, Jalan Raja Muda Abdul Aziz, Kuala Lumpur 50300, Malaysia; nooribrahim@ukm.edu.my; 7 Internal Medicine and Geriatric Department, Pusat Perubatan Universiti Kebangsaan Malaysia, Jalan Yaacob Latif, Bandar Tun Razak, Batu 9 Cheras, Kuala Lumpur 56000, Malaysia; drhazlina2013@gmail.com; 8 Centre for Healthy Ageing and Wellness (H-CARE), Faculty of Health Sciences, Universiti Kebangsaan Malaysia, Jalan Raja Muda Abdul Aziz, Kuala Lumpur 50300, Malaysia; m.zulamin@ukm.edu.my

**Keywords:** psychological distress, older adults, COVID-19, food insecurity, nutrient intake

## Abstract

This study aimed to investigate the impact of food insecurity and poor nutrient intake on the psychological health of middle-aged and older adults during the COVID-19 pandemic. A sub-sample of 535 individuals aged 52 years and above, from the earlier cohort and interventional studies (*n* = 4) from four selected states in Peninsular Malaysia, were recruited during the COVID-19 outbreak (April to June 2020). Telephone interviews were conducted by trained interviewers with a health sciences background to obtain participants’ information on health status, physical activity, food security, and psychological health (General Health Questionnaire-12; normal and psychological distress). Univariate analyses were performed for each variable, followed by a logistic regression analysis using SPSS Statistics version 25.0. Results revealed food insecurity (OR = 17.06, 95% CI: 8.24–35.32, *p* < 0.001), low protein (OR = 0.981, 95% CI: 0.965–0.998, *p* < 0.05), and fiber intakes (OR = 0.822, 95% CI: 0.695–0.972, *p* < 0.05) were found to be significant factors associated with the psychological distress group after adjusting for confounding factors. The findings suggested that food insecurity and insufficiencies of protein and fiber intakes heightened the psychological distress during the COVID-19 pandemic. Optimal nutrition is vital to ensure the physical and psychological health of the older population, specifically during the current pandemic.

## 1. Introduction

The coronavirus (COVID-19) outbreak has led to unprecedented hazards to mental health and threatened the overall well-being of the population [1]. Along with uncertainties and even fear associated with the virus outbreak, citizens have to cope with the distressing experience of the loss of freedom and separation from family members and friends due to lockdowns [2]. The rapid transmission of the COVID-19 pandemic outbreak, higher mortality rate, and prolonged isolation due to lockdowns may result in an increased number of psychological problems including post-traumatic stress symptoms, psychological distress, depressive symptoms, stress, and emotional disturbance [2,3]. It is a well-known fact that older people have a higher risk of infection and mortality due to COVID-19 [4], but the information related to their psychological status and risk of psychological distress is limited.

Apart from the impact on mental health, the COVID-19 pandemic has led to a global economic crisis due to lockdowns and yielded negative economic growth, with massive public health and social safety costs [5]. The disruption caused by the COVID-19 pandemic has also resulted in food insecurity among many people [6]. Food insecurity is a disruption in food intake or eating patterns with limited access to sufficient, nutritious food due to a lack of money and other resources [7]. Numerous surveys have documented the unprecedented levels of food insecurity since the start of the pandemic [8,9,10]. Food insecurity is a stressful experience and has been associated with numerous detrimental physical and mental health outcomes, and it thus becomes an increasing concern [11]. In addition, those impacted by poverty or food insecurity are likely to experience additional resource-related hardships, that in turn, contribute to poor nutrition, health, and disease management [12,13].

Nutrition, physical activity, and socialization are important contributors to the enhancement of physical and mental resilience, especially among the older population. However, the COVID-19 pandemic has disturbed all these factors due to physical distancing and social activities restriction, leading to physical frailty, sarcopenia, and malnutrition [14]. Social isolation can expose older adults to increased nutritional risk due to factors such as socioeconomic insecurity, which could affect food acquisition and the needs for support in daily tasks and meal preparations [15]. Besides, nutrition and food intakes play significant roles in the psychological health of older adults [16], which warrants further investigation due to limited information. Understanding factors related to psychological distress during the COVID-19 outbreak among older adults is crucial to maintaining their psychological well-being. Earlier, we have revealed that most of the Malaysian middle-aged and older adults have good psychological health and adopt positive coping mechanisms during this crisis [17]. Therefore, in this study, we further investigated the impact of food insecurity and poor nutrient intake on the psychological health of older adults during this pandemic outbreak.

## 2. Materials and Methods

### 2.1. Study Design

This study was designed as a cross-sectional study, using data obtained from the earlier cohort Long-term Research Grant Scheme-Towards Useful Aging (LRGS TUA) [18] and several interventional studies [19,20,21] as its baseline. A sub-sample of 535 individuals aged 52 years and above (men = 238, women = 297) from four states with the highest older adult populations, namely Selangor, Perak, Kelantan, and Johor (representing central, northern, eastern, and southern regions of Malaysia), were recruited in this study to assess their psychological health during the nationwide Movement Control Order (MCO) period due to the COVID-19 outbreak. MCO was referred to as a partial lockdown, an action enforced by the Malaysian Government to contain the transmission of the COVID-19 virus, which prohibited mass movements and gatherings, travel restrictions, as well as the closure of all business premises, private premises, and learning institutions [22]. Participants were recruited using a purposive sampling method. The inclusion criteria included Malaysian citizens aged 52 years and above with normal hearing, able to converse in either the Malay, English, Chinese, or Tamil languages with no documented major psychiatric illnesses or mental disorders. The protocol for this study was approved by the Medical Research and Ethics Committee of Universiti Kebangsaan Malaysia (UKM1.21.3/244/NN-2018-145). Verbal informed consent was acquired from all participants prior to their enrollment after elucidating the fact that participation was voluntary and that anonymity and confidentiality would be strictly maintained. Additional permission for recording the call was also taken from every participant.

### 2.2. Data Collection

The information obtained from previous databases were sociodemographic status, anthropometric measurements, functional status, depression, and nutrient intakes of the participants. Amid the nationwide Movement Control Order (MCO) period, the telephone interviews were conducted from April to June 2020 by six trained interviewers with a health sciences background (nutrition, dietetics, and physiotherapy) to obtain participants’ self-reported health status, physical activity, food security, and general psychological health. They were employed for the study and had regular monitoring and retraining. The interview took about 20 to 30 min per participant. Interviewers attempted three phone calls to reach the participants. Each response to the questionnaire was immediately entered into an electronic database. All interviews were recorded for quality control purposes and data analysis improvement. Any uncompleted questions were verified with participants through follow-up phone interviews. In data quality parameters, the details on the data-verification of pattern, branching, output format and also data accuracy, and cleaning as per specifications were thoroughly checked.

### 2.3. Study Instruments

#### 2.3.1. Sociodemographic Information and Health Status

The sociodemographic and health variables obtained included age, gender, ethnicity, living arrangement, marital status, educational level, years in formal education, occupation, household income, and smoking status. Besides that, self-reported information on certain diseases (cardiovascular disease, hypertension, lung disease, liver disease, diabetes mellitus, gastritis, kidney disease, cancer) diagnosed by doctors in the prior years were also recorded.

#### 2.3.2. Anthropometric Measurements

The anthropometric measurements were recorded as the secondary data. The data included standing height, weight, mid-upper arm circumference, waist circumference, hip circumference, and calf circumference, and they were assessed according to standard protocols [23]. The Body Mass Index (BMI, kg/m^2^) was calculated as the body weight in kilograms divided by the squared standing height in meters. The body circumferences were measured using a non-extensible and flexible plastic measuring tape.

#### 2.3.3. Instrumental Activities of Daily Living (IADL)

The Lawton Instrumental Activities of Daily Living Scale (IADL) was used to assess independent living skills. It was developed by Lawton and Brody in 1969 [24]. The IADL questionnaire used in this study was translated into the Malay language and contained seven items related to the ability to use a phone, shopping, doing housework, managing finances, travel, food preparation, and taking one’s own medications. The Malay version of IADL has been validated by a previous study, and the Cronbach’s alpha coefficient for internal consistency was 0.838 [25].

#### 2.3.4. Geriatric Depression Scale (GDS)

The Geriatric Depression Scale (M-GDS) was used to assess potential depressive symptoms, a self-rated scale consisting of 14 items with dichotomous responses of “yes” or “no” in reference to how participants felt on the day the questionnaire was administered. The original GDS developed by Yesavage and Sheikh [26] has been proven to have high sensitivity and specificity. It is a valid and reliable instrument with Cronbach’s alpha values ranging from 0.88 to 0.91. In this study, we adopted Malay-translated GDS (M-GDS-15), which was validated and shown to have a good internal consistency (Cronbach’s alpha, 0.84) [27]. The higher score of M-GDS-15 indicated more depressive symptoms among our participants.

#### 2.3.5. Dietary Intake

The dietary intake was obtained using the Dietary History Questionnaire (DHQ) [28]. Participants were interviewed individually to obtain their usual food and drink intake within a week. The amount of food intake was measured using a food album and household measurements such as cups, glasses, teaspoons, tablespoons, and bowls of various sizes to obtain a precise food intake of subjects. The nutrient intake was analyzed using the Nutritionist Pro^TM^ software. The output from the software was then exported into the Excel database.

#### 2.3.6. Physical Activity

The validated Malay version of the International Physical Activity Questionnaire- Short Form (IPAQ-SF) was used to measure the physical activity of the participants [29]. The physical activity level and intensity were calculated in metabolic equivalent task minutes per week (MET-minutes/week), determining the duration (in minutes) and the number of days (in one week) of engagement in three levels of activities (walking, moderate-intensity, and high-intensity activities). This was conducted across a comprehensive set of domains (leisure time, work-related and transport-related physical activities, domestic and gardening activities) in the past seven days. METs or metabolic equivalent is a unit used to estimate the amount of oxygen used by the body during physical activity. A MET-minutes/week was computed by multiplying the MET score of activity (3.3 for walking, 4.0 for moderate-intensity, and 8.0 for vigorous-intensity) by the minutes and the days (or the sessions) of engagement [30]. The total score of physical activity was used in this study.

#### 2.3.7. Food Security

Food security status was assessed using the United States Department of Agriculture (USDA) Household Food Security Survey: a Six-Item Short Form developed by researchers at the National Center for Health Statistics [31]. The sum of affirmative responses to the six questions in the module was the household’s raw score on the scale and categorized as follows: (1) 0–1 = high or marginal food security; (2) 2–4 = low food security; (3) 5–6 = very low food security. In this study, the categories of low and very low food securities were combined into a category referred to as food insecurity.

#### 2.3.8. Psychological Distress

The Short General Health Questionnaire-12 (GHQ-12) consisting of 12 questions and was used to assess the current psychological state of the participants [32]. Each question had four responses. The participants’ answers were scored as 0-0-1-1 based on their responses. The total score was determined by adding the score obtained for each answer in the questionnaire. The final scale ranged from 0 to 12, with higher scores indicating higher short-term psychological distress. Based on the GHQ-12 guidelines, scores of 4 and above were indications of psychological distress. This scale had good internal consistency, with Cronbach’s alpha coefficient at 0. 93. In this study, participants with scores below 4 were grouped as normal, while those with scores of 4 and above were categorized as psychological distress.

### 2.4. Statistical Analysis

All the analyses were conducted using the IBM Statistical Package for Social Sciences (IBM SPSS), version 25.0 (SPSS Inc., Chicago, IL, USA). The statistical significance level was set at alpha <0.05 for all the tests performed. Descriptive and frequency analyses were executed for the prevalence of psychological health status (normal and psychological distresses). Comparisons of the sociodemographic factors, health status, anthropometric measurements, physical activity, functional status, depression scale, food security, and dietary intake between the normal and the psychological distress groups were analyzed using Chi-square test for the categorical variables and independent *t*-tests for the continuous variables. The results were presented as *n* (%) and mean ± standard deviation for normally distributed data.

A binary logistic regression (BLR) was performed to determine the factors associated with psychological distress in a multivariate model. Firstly, all the significant variables in the univariate analysis were categorized into two different groups according to (1) sociodemographic and medical status; (2) anthropometric measurements, functional depression status, and physical activity. Then, a hierarchical binary logistic regression was conducted for the two categories. Variables that appeared significant (*p* < 0.05) in each model were selected as the confounding factors into the final binary logistic model (food security and nutrient intake). The significant variables in the final model were those factors that were associated with psychological distress among the study population.

## 3. Results

Approximately, of the 535 participants included in the analysis (response rate = 81.1%), 88.0% of participants had normal psychological health, whereby only 12% of the participants reported to have psychological distress during this pandemic.

As stated in Table 1, the mean age in this study was 71.18 ± 5.72 years old, and those in the psychological distress group (72.50 ± 6.00 years) were older than the normal group (71.00 ± 5.66 years) (*p* < 0.05). The gender distribution of this study consisted of 55.5% women and 44.5% men (*p* > 0.05). The majority of the participants were Malays (59.6%), followed by Chinese (35.0%), and Indians (5.4%) (*p* < 0.01). In addition, 90.9% of the participants lived together with their family (*p* < 0.05). The psychological distress group had a significantly lower mean year of education (5.14 ± 3.75 years) compared to the normal group (7.23 ± 4.34 years) (*p* < 0.001). However, marital status, living arrangement, household income, occupation, and smoking habit did not show any significant difference between the normal and the psychological distress groups (*p* > 0.05). Besides that, participants in the psychological distress group were reported to have higher prevalence of gastritis (21.0%) compared to the normal group (10.8%) (*p* < 0.01).

As shown in Table 2, no significant differences were presented between the normal and the psychological distress groups in the anthropometry and IADL score (*p* > 0.05). However, participants in the psychological distress group (3.25 ± 2.40) had a higher risk of depressive symptoms as compared to the normal group (2.55 ± 2.43) (*p* < 0.05). Moreover, those in the normal group (537.42 ± 327.80 MET-min/week) had a higher mean value of physical activity compared to participants in the psychological distress group (144.52 ± 187.65 MET-min/week) (*p* < 0.001).

Table 3 presents that the participants in the psychological distress group (71.9%) had a higher prevalence of food insecurity as compared to the normal group (7.0%) (*p* < 0.001), with overall prevalence of 14.8%. Besides that, about 18.9% of adults experienced food quantity insufficiency, 11.4% had food variety insufficiency, 11.0% practiced reduced size of the meal, and 2.2% skipped the main meal. With respect to the dietary intake, the energy intakes of the psychological distress group appeared to be lower (1383 ± 301 kcal/day) than those of normal group (1407 ± 326 kcal/day), but was not significantly different (*p* > 0.05). The means for nutrient, including protein, cholesterol, and fiber intakes, were significantly lower among the psychological distress group than the normal group (*p* < 0.001). Furthermore, the intakes of beta-carotene, vitamin K, and copper were also lower in the psychological distress group as compared to the normal group (*p* < 0.05). The intake of other nutrients such as vitamin A, beta-carotene, vitamin C, thiamin, folate, iron, magnesium, and copper were reported lower among the psychological distress group than the normal group, with non-significant statistical difference (*p* > 0.05).

Table 4 shows the results of the binary logistic regression (BLR) analysis after adjusted for age, ethnic, years of education, gastritis, physical activity, and depression. Food insecurity (Adj OR = 17.06, 95% CI: 8.24–35.32, *p* < 0.001), low protein intakes (Adj OR = 0.981, 95% CI: 0.965–0.998, *p* < 0.05), and fiber intakes (Adj OR = 0.822, 95% CI: 0.695–0.972, *p* < 0.05) were found to be significant factors associated with the psychological distress group.

## 4. Discussion

Generally, only 12.0% of the Malaysian middle-aged and older adults in this study were reported to have psychological distress during this COVID-19 pandemic. This is surprising as this pandemic may have a potentially beneficial impact on the mental health of the older population in the Malaysian setting. Earlier studies reported that 35–51.6% of adults in China experienced psychological distress, which is three to four times higher than the prevalence reported in this study [33,34]. However, the difference in the studied population and the tools used to describe psychological distress may explain the prevalence discrepancies between these studies. Previous study has used the COVID-19 Peritraumatic Distress Index (CPDI), which specifically could inquire about the psychological distress during this pandemic crisis [33]. On the other hand, the GHQ-12 used in this study has high sensitivity and specificity, where some items in this instrument, namely “feeling unhappy and depressed”, “lost much sleep”, and “under strain” strongly indicated anxiety and depressive symptoms [35]. Depression, unhappiness, and sleep disturbance in response to stressful life events was to be expected, especially during this COVID-19 pandemic due to drastic changes in daily routines and prolonged measures of social confinement and isolation [36]. Therefore, the items in the GHQ-12 highly represented the factors of psychological distress, suggesting that the GHQ-12 was also a good tool for assessing psychological health during the COVID-19 pandemic.

Finding a lower prevalence of distress among the older population might reflect their resilience from having overcome adversities and experiencing fewer daily disruptions than the younger groups [37]. It was noted that most individuals in this study stayed together with their family members. The movement control order (MCO), a partial lockdown, could have brought families together, strengthening family bonding, and eventually improving family support, which are vital in facing a crisis [38]. However, these situations were contradictory to their counterparts, where people with psychological distress had higher prevalence of living alone in the recent study. It is reported that older adults living alone were more likely to be depressed and have poor mental health [39]. Therefore, it is likely that Malaysian middle-aged and older adults could have maintained or improved their psychological health by having strong family support to face this pandemic crisis.

In this current study, food insecurity was found to be an important risk factor of psychological distress among middle-aged and older adults during the movement control order due to the COVID-19 pandemic. In accordance with the present results, previous studies have demonstrated that food insecurity contributed to the development of stress, anxiety [40], depression, and psychological distress [41]. Based on the previous studies [40,41,42], food insecurity and mental health are already in a complex relationship and the COVID-19 pandemic certainly will affect the psychosocial well-being of older adults. It has been postulated that poor nutrition, stress, and shame about having food insecurity may lead to increased risk of mental health problems, particularly for older adults [40,43]. However, the reported prevalence of food insecurity levels among older adults were lower (14.8%) compared to previous studies, where 23.1% of Jordanian [44] and 19.5% to 19.7% of Malaysian older adults experienced food insecurity [45,46]. Besides, findings from the Malaysian Adult Nutrition Survey (MANS) revealed that about 25.5% and 21.9% of the Malaysian population experienced food quantity and food variety insufficiency, which were higher than that reported in this study [47]. The stigma and the shame associated with food insecurity of not being able to provide adequately for their families commonly lead to underreporting by older people [48]. Another possible explanation for this might be that most of them were living together with their family members during MCO, and that perhaps the children are the ones that provided all the food and prepared the meals for their parents since they were working from home.

Moreover, low intake of protein is associated with psychological health among middle-aged and older people. Protein intake of an individual with psychological distress was relatively lower than the protein recommendation for middle-aged and older adults (men: 61 g/day, women: 52 g/day; older men: 58 g/day, older women: 50 g/day) [49]. This could be explained by the fact that most of them who experienced food insecurity tended to consume a poorer quality diet and lower level of essential nutrients [50]. Insufficiencies of protein and essential amino acid, particularly tryptophan, resulted in decreased neurotransmitter synthesis, which plays an important role in mood alleviation, satiety, and sleep regulation [51]. Besides, this may cause more harm by reducing proteins such as brain-derived neurotrophic factor (BDNF) and up-regulating of the stress response, and the immune and oxidative systems [52]. Clearly, it is important to prevent food insecurity and to improve the diet quality of adults, as both factors are critical to their mental health.

In addition, adults with psychological distress tended to consume low amounts of fiber and did not even achieve the recommendation for all age groups (20–30 g/day) [49]. A systematic review has revealed that low intake of dietary fiber such as fruits and vegetables had influences on various aspects of mental health, including mental well-being, quality of life, mood, stress, distress, and depression [53]. In fact, the potential of dietary fiber in the reduction of depressive symptoms and improvement of psychological well-being has been reported in previous intervention studies [54,55]. A possible mechanism underlying this relationship might be due to higher intake of fiber, which was able to reduce the production of inflammatory cytokines. These cytokines are known as strong contributors to increase depression severity and eventually increase the production of serotonin and dopamine neurotransmitters [56]. Nevertheless, future investigations are required to further clarify this relationship.

A few limitations in the present study should be acknowledged when interpreting the findings. Firstly, all items and measures were based on self-report, which were susceptible to recall bias and social desirability. This would likely lead to underreporting of the measures, particularly food security due to feelings of shame and stigma. Nevertheless, self-reporting might be the most convenient way to capture subjective views through telephone interviews. Secondly, in this study, we used previous databases to obtain baseline information of the participants, whereby missing data on target variables was a potential limitation. However, analysis using imputed data for missing values in nutrient intake and anthropometric measurements have been used in this study. Thirdly, other confounding factors might still exist; for example, quality of life, biochemical parameters, and cognitive status, although we adequately controlled for potential cofounders. Despite these limitations, these findings added valuable insights on the importance of improvement of food security and specific nutrients, particularly protein and fiber intakes in alleviating psychological distress among middle-aged and older adults, especially during this pandemic crisis.

## 5. Conclusions

In conclusion, the findings emphasized the food insecurity and the insufficiencies of protein and fiber intakes associated with psychological distress among Malaysian middle-aged and older adults during movement control order due to the COVID-19 pandemic. Therefore, this study could be a stepping stone for establishing programs and prevention strategies to reduce or to manage psychological distress among middle-aged and older adults, particularly during this pandemic crisis.

## Figures and Tables

**Table 1:** Sociodemographic data and health status between normal and psychological distress participants [presented as mean ± standard deviation and number of participants (%)].

Parameter	Total (*n* = 535)	Normal (*n* = 471)	Psychological Distress (*n* = 64)	*p*-Value
**Demographic**				
Age (year)	71.18 ± 5.72	71.00 ± 5.66	72.50 ± 6.00	0.049 *
Gender				
Men	238 (44.5)	205 (43.5)	33 (51.6)	0.231
Women	297 (55.5)	266 (56.5)	31 (48.4)	
Ethnic:				
Malay	319 (59.6)	268 (56.9)	51 (79.7)	0.001 **
Chinese	187 (35.0)	178 (37.8)	9 (14.1)	
Indian	29 (5.4)	25 (5.3)	4 (6.3)	
Marital status:				
Married	185 (34.6)	167 (35.5)	18 (28.1)	0.266
Single/divorced	350 (65.4)	304 (64.5)	46 (71.9)	
Number of households	3.31 ± 1.85	3.31 ± 1.86	3.31 ± 1.83	0.987
Living arrangement:				
Living alone	46 (9.1)	37 (8.4)	46 (9.1)	0.040 *
Living together with family	459 (90.9)	404 (91.6)	459 (90.9)	
Education level:				
No formal education	63 (12.0)	53 (11.5)	10 (15.6)	0.410
Formal education	461 (88.0)	407 (88.5)	54 (84.4)	
Years of education	6.98 ± 4.33	7.23 ± 4.34	5.14 ± 3.75	<0.001 ***
Household income (RM)	1863.16 ± 4863.42	1945.74 ± 5127.47	1296.72 ± 2305.15	0.319
Occupation:				
Not working/Housewife	343 (64.1)	306 (65.0)	37 (57.7)	0.269
Pensioner/Working	192 (35.9)	165 (35.0)	27 (42.2)	
Smoking status:				
Not smoking	380 (75.2)	336 (76.2)	44 (68.8)	0.215
Smokers/Ex-smokers	125 (24.8)	105 (23.8)	20 (31.2)	
Health status:				
CVD	59 (11.0)	50 (10.6)	9 (14.1)	0.397
HPT	303 (56.6)	267 (56.7)	36 (56.3)	0.947
Lung disease	4 (0.7)	4 (0.8)	0 (0.0)	0.459
Liver disease	2 (0.4)	2 (0.4)	0 (0.0)	0.601
DM	155 (29.0)	137 (29.1)	18 (28.1)	0.874
Gastritis	67 (12.5)	51 (10.8)	16 (25.0)	0.004 **
Kidney disease	15 (2.8)	15 (3.2)	0 (0.0)	0.236
Cancer	15 (2.8)	13 (2.8)	2 (3.1)	0.698

*** *p* < 0.001; ** *p* < 0.01; * *p* < 0.05—significant using Pearson Chi-square for categorical data and Independent *t*-test for numerical data. Abbreviations: CVD = Cardiovascular disease; DM = Diabetes mellitus; HPT = Hypertension; RM = Ringgit Malaysia.

**Table 2:** Anthropometry, physical activity, depression and functional status between normal and psychological distress participants [presented as mean ± standard deviation and number of participants (%)].

Parameter	Total (*n* = 535)	Normal (*n* = 471)	Psychological Distress (*n* = 64)	*p*-Value
Anthropometry:				
BMI (kg/m^2^)	25.50 ± 4.53	25.55 ± 4.58	25.15 ± 4.18	0.509
BMI categories (WHO):				
Underweight	19 (3.8)	15 (3.5)	4 (6.5)	0.253
Normal	232 (46.8)	207 (47.7)	25 (40.3)	
Overweight	168 (33.9)	142 (32.7)	26 (41.9)	
Obesity	77 (15.5)	70 (16.1)	7 (11.3)	
MUAC (cm)	28.23 ± 3.99	28.22 ± 4.04	28.29 ± 3.73	0.901
Waist circumference (cm)	87.93 ± 11.56	88.16 ± 11.74	86.56 ± 10.36	0.308
Hip circumference (cm)	97.98 ± 9.92	98.06 ± 9.97	97.51 ± 9.63	0.683
Calf circumference (cm)	34.85 ± 15.26	35.07 ± 16.42	33.56 ± 3.49	0.468
IADL	13.17 ± 1.77	13.23 ± 1.64	12.80 ± 2.38	0.071
GDS	2.65 ± 2.43	2.55 ± 2.43	3.25 ± 2.40	0.032 *
Physical activity (MET-min/week)	490.33 ± 339.16	537.42 ± 327.80	144.52 ± 187.65	<0.001 ***

*** *p* < 0.001; * *p* < 0.05—significant using Pearson Chi-square for categorical data and Independent *t*-test for numerical data. Abbreviation: BMI = Body mass index; MUAC = Mid-upper arm circumference; IADL = Instrumental activities of daily living; GDS = Geriatric depression scale; WHO = World Health Organization.

**Table 3:** Food security and nutrient intakes between normal and psychological distress participants [presented as mean ± standard deviation and number of participants (%)].

Parameter	Total (*n* = 535)	Normal (*n* = 471)	Psychological Distress (*n* = 64)	*p*-Value
Food security:				
High	456 (85.2)	438 (93.8)	18 (28.1)	<0.001 ***
Low	79 (14.8)	33 (7.0)	46 (71.9)	
Nutrient Intake:				
Energy (kcal/day)	1404 ± 323	1407 ± 326	1383 ± 301	0.607
Protein (g/day)	72.91 ± 42.76	75.40 ± 44.90	55.73 ± 14.08	<0.001 ***
Carbohydrate (g/day)	163.43 ± 63.50	161.90 ± 65.72	174.03 ± 44.26	0.075
Fat (g/day)	50.85 ± 15.57	50.73 ± 15.54	51.66 ± 15.90	0.675
Cholesterol (mg/day)	206.48 ± 180.51	215.68 ± 188.58	152.25 ± 108.79	<0.001 ***
Fiber (g/day)	4.49 ± 2.58	4.61 ± 2.65	3.63 ± 1.78	<0.001 ***
Vit A (RE/day)	630.86 ± 256.93	636.16 ± 257.58	599.65 ± 253.08	0.322
Beta-Carotene (µg/day)	2379.08 ± 1242.40	2467.04 ± 1228.09	1860.60 ± 1209.35	0.001 **
Vit C (mg/day)	90.29 ± 57.27	92.58 ± 56.77	76.73 ± 58.83	0.053
Vit D (µg/day)	0.13 ± 0.42	0.13 ± 0.41	0.20 ± 0.53	0.451
Vit E (mg/day)	18.01 ± 86.66	14.31 ± 79.09	39.81 ± 120.75	0.129
Thiamin (mg/day)	0.62 ± 0.92	0.64 ± 0.99	0.56 ± 0.17	0.580
Riboflavin (mg/day)	0.86 ± 0.27	0.86 ± 0.26	0.88 ± 0.32	0.633
Niacin (mg/day)	8.58 ± 2.46	8.55 ± 2.51	8.79 ± 2.09	0.492
Pyridoxine (mg/day)	0.77 ± 0.36	0.78 ± 0.36	0.71 ± 0.34	0.182
Folate (µg/day)	87.34 ± 61.78	88.85 ± 56.84	78.42 ± 85.42	0.239
Cobalamin (µg/day)	3.51 ± 2.83	3.48 ± 2.91	3.71 ± 2.28	0.575
Vit K (µg/day)	20.74 ± 47.24	23.00 ± 50.13	7.37 ± 19.48	<0.001 ***
Potassium (mg/day)	1268.06 ± 342.74	1268.69 ± 342.55	1264.37 ± 346.90	0.930
Calcium (mg/day)	350.71 ± 212.53	351.68 ± 204.04	343.97 ± 265.93	0.798
Iron (mg/day)	14.68 ± 24.04	15.29 ± 25.64	10.44 ± 3.20	0.155
Phosphorus (mg/day)	758.66 ± 221.39	757.20 ± 220.44	767.28 ± 228.72	0.751
Magnesium (mg/day)	110.06 ± 66.51	111.92 ± 70.16	99.10 ± 37.05	0.179
Zinc (mg/day)	4.22 ± 11.31	4.48 ± 12.20	2.68 ± 1.29	0.266
Copper (mg/day)	0.46 ± 0.27	0.47 ± 0.28	0.39 ± 0.15	0.030 *

*** *p* < 0.001; ** *p* < 0.01; * *p* < 0.05—significant using Pearson Chi-square for categorical data and Independent *t*-test for numerical data. Abbreviation: Vit = Vitamin.

**Table 4:** Factor associated with psychological distress.

Parameters	Estimate	Standard Error	OR (95% CI)	*p*-Value
**Food security:**				
High				
Low (ref)	2.836	0.371	17.06 (8.24, 35.32)	< 0.001
**Nutrient intake:**				
Protein (g/day)	−0.019	0.009	0.981 (0.965, 0.998)	0.027 *
Cholesterol (mg/day)	−0.002	0.002	0.998 (0.995, 1.001)	0.175
Fiber (g/day)	−0.196	0.086	0.822 (0.695, 0.972)	0.022 *
Beta-Carotene (µg/day)	0.000	0.000	1.000 (1.000, 1.000)	0.767
Vitamin K (µg/day)	−0.003	0.007	0.997 (0.984, 1.010)	0.627
Copper (mg/day)	−1.033	0.967	0.356 (0.054, 2.368)	0.285

* *p* < 0.05—significant using Binary Logistic Regression (BLR) with psychological distress as the reference group (0 = normal, 1 = psychological distress). This model was adjusted for age, ethnicity, living arrangement, years of education, gastritis, physical activity, and depression.

## Data Availability

The data presented in this study are available on request from the corresponding author. The data are not publicly available due to confidentiality concerns.
